# Interactive seminars or small group tutorials in preclinical medical education: results of a randomized controlled trial

**DOI:** 10.1186/1472-6920-10-79

**Published:** 2010-11-13

**Authors:** Zuzana de Jong, Jessica AB van Nies, Sonja WM Peters, Sylvia Vink, Friedo W Dekker, Albert Scherpbier

**Affiliations:** 1 Department of Rheumatology, Leiden University Medical Center, P.O. Box 9600, 2300 RC Leiden, The Netherlands; 2 Center for Educational Expertise in Medicine, Leiden University Medical Center, P.O. Box 9600, 2300 RC Leiden, The Netherlands; 3 Institute for Medical Education, Faculty of Health, Medicine and Life Sciences, Maastricht University, Maastricht, The Netherlands

## Abstract

**Background:**

Learning in small group tutorials is appreciated by students and effective in the acquisition of clinical problem-solving skills but poses financial and resource challenges. Interactive seminars, which accommodate large groups, might be an alternative. This study examines the educational effectiveness of small group tutorials and interactive seminars and students' preferences for and satisfaction with these formats.

**Methods:**

Students in year three of the Leiden undergraduate medical curriculum, who agreed to participate in a randomized controlled trial (RCT, n = 107), were randomly allocated to small group tutorials (n = 53) or interactive seminars (n = 54). Students who did not agree were free to choose either format (n = 105). Educational effectiveness was measured by comparing the participants' results on the end-of-block test. Data on students' reasons and satisfaction were collected by means of questionnaires. Data was analyzed using student unpaired t test or chi-square test where appropriate.

**Results:**

There were no significant differences between the two educational formats in students' test grades. Retention of knowledge through active participation was the most frequently cited reason for preferring small group tutorials, while a dislike of compulsory course components was mentioned more frequently by students preferring interactive seminars. Small group tutorials led to greater satisfaction.

**Conclusions:**

We found that small group tutorials leads to greater satisfaction but not to better learning results. Interactive learning in large groups might be might be an effective alternative to small group tutorials in some cases and be offered as an option.

## Background

In recent decades, renovations of medical curricula have often been accompanied by the introduction of small group learning. The effects of this educational format have been examined in many studies, mostly within contexts of problem-based learning [1]. A recent review of cognitive and motivational effects of small group tutorials [2] showed that small study groups foster interactive learning and positive cognitive effects, such as activation of prior knowledge, recall of information, individual and collaborative knowledge construction, and cognitive conflicts leading to conceptual change [3-6]. Small group learning was also reported to have a direct positive effect on students' motivation to learn [7,8] and motivation has been shown to play a central role in promoting group productivity, elaboration of knowledge, and interaction in different settings. Finally, interactive learning has been evaluated more positively than formal lecturing by medical students [9] and medical professionals [10,11] alike.

Despite the known beneficial effects of small group learning, practical reasons, notably increasing student numbers, have necessitated deviations from the original concept with regard to set-up and group size. For example, although groups of 8-10 students with 1 tutor [12] are generally considered optimal, increases to as many as 15 students per tutor have been reported and further increases are likely [13]. It may be prudent, therefore, to look for adequate but less resource intensive alternatives. Evidence with regard to such alternatives may also be valuable, in light of doubts that have been raised regarding the effectiveness of small group tutorials. Indeed some studies have pointed out that, in practice, small group tutorial is not always experienced as effective [14-18] and others have suggested that there may be some incongruence between educational theory and practice in this respect [6,19].

Interactive seminars may offer a good alternative to small group learning, because they also enable active participation and interaction but are less of a drain on resources, as they can accommodate quite large groups of students. In contrast to formal lecturing, interactive seminars make use of several techniques to promote interaction, such as questioning the audience, interactive computer systems providing learners with immediate, anonymous feedback on their knowledge, clinical cases, debates between students presenting different opinions, films or videotapes or other audiovisual aids, and effective presentation skills. Interactive seminars promote active involvement and increased attention and motivation of students. It can "facilitate problem-solving and decision-making" and provide direct feedback to teachers and learners [20]. In addition, compared to formal lectures, it increases students' and teacher's satisfaction with the teaching format [21,22,5]. However, it remains to be demonstrated whether the educational effectiveness of interactive seminars is comparable to that of small group tutorials in terms of student learning and satisfaction. Therefore we examined the differential impact of small group tutorials and interactive seminars.

This article reports on

1. The educational effectiveness of small group tutorials versus interactive seminars

2. Students' reasons for preferring small group tutorials or interactive seminars

3. Satisfaction with the format of their choice of students who were free to choose between small group tutorials and interactive seminars.

## Methods

### Setting

The curriculum of Leiden Medical School, the Netherlands, consists of four preclinical and two clinical years and was recently reconstructed in accordance with the Calgary model of Clinical Presentations[23]. The preclinical semesters consist of 3 to 6-week blocks featuring different educational formats, such as formal lectures, self-directed learning and small group tutorials of 15 students and 1 tutor, but not interactive seminars. None of the educational formats is obligatory. Our comparison of small group tutorials and interactive seminars was conducted in the block on MusculoSkeletal Problems (MSP) in the final 3 weeks of Year 3. In this block, students learn to apply structured clinical reasoning in dealing with problems of the musculoskeletal system. Most students who participate in the block sit the end-of-block test on the last day of the block. According to the Medical School rules, students who undertake the block are not obliged to sit the test at that particular moment and are free to choose an other opportunity.

### Recruitment of participants

Two months before data collection started, all students who were eligible to undertake the MSP block (potential participants) were asked to participate in a randomized controlled trial (RCT). Those who agreed to participate (RCT participants) were randomly allocated to small group tutorials or interactive seminars by an independent person (department administrator not involved in the study) who was not aware of the students' characteristics or test results. No incentives were provided. The RCT participants were urged to attend every session and their attendance was monitored. The students who did not participate were offered a choice between small group tutorials and interactive seminars. Their data were only used for this study if they completed at least one of two questionnaires (non-RCT participants). Students who did not complete any of the questionnaires were regarded as non-responders.

Although medical ethics committees in Dutch academic medical centres are currently not required to evaluate this type of study, an ethical procedure was agreed on with the academic hospital's medical ethics advisor. This meant that students were informed that their data (questionnaires and grades) would be anonymized. They were also invited to express any disagreement with the procedure and given the assurance that, if they disagreed, their data would be removed from the database. No disagreement was expressed by any of the students. Because of the ethical reasons, available data of the students who did not fill in any of the questionnaires (non-responders) were not used for analysis.

### Educational formats

During the 3-week MSP block, students can attend in addition to formal lectures (on average 8 hours each week) and real patient learning-practical's (1 1/2 hours each week), three small group tutorials or three interactive seminars, one on rheumatology and two on orthopaedics. Both, small group tutorials and interactive seminars, were designed by highly experienced teachers who had participated in a variety of faculty development activities. These two educational formats are the subject of this study.

#### Small group tutorials

Weekly 2-hour tutorials were a regular component of the 3-week MSP block. Average group size was 15 students with one tutor, and the tutors were residents in orthopaedic surgery or rheumatology who had attended special tutor training, consisting of one hour devoted to 'How to facilitate interaction in a tutorial' and 1 hour on case content. In addition, all tutors received a tutor guide written by the block coordinator to assure that the same important aspects of the case under analysis would be discussed in all groups. During the block sessions students first collaboratively analyzed clinical cases concerning musculoskeletal complaints and formulated differential diagnoses. Subsequently, the cases were systematically analyzed for missing signs and symptoms which could support a diagnosis, and diagnostic procedures and therapeutic options were discussed for each diagnostic alternative. No audiovisual methods were used.

#### Interactive seminars

The interactive seminars were developed as an alternative to the small group tutorials. They were also held weekly, lasted 2 hours, and the clinical cases addressed were identical to those tackled by the small groups. The cases were presented by a senior staff member, trained in interactive lecturing, to a group of some 80 students and systematically analyzed using the same method as the one employed by the small groups. Incomplete case handouts were distributed at the start of the session and students were stimulated to complete these during the seminar. The participants were actively encouraged to interact with each other and with the teacher. The lecturer frequently questioned the audience, using alternatively straightforward questions, surveying the class to identify audience needs and interests, brainstorming and rhetorical questions [20]. Audiovisual techniques, such as videotapes and PowerPoint presentations, were used to illustrate cases, clarify the problems and promote interaction.

### Data collection

Questionnaire A collected baseline data at the start of the study and questionnaire B was administered after the block test. An educationalist (SV), student representatives participating in the block (JvN and SP) and the block coordinator (ZdJ) collaboratively developed the two questionnaires and tested their feasibility.

All RCT and non-RCT participants received questionnaire A by email three weeks before the block and one reminder shortly before the block started. The questionnaire items asked about gender and average number of hours per week (< 20 hours, 21-30 hours, >30 hours) spent on didactic lectures, small group tutorials, and independent study during the preceding blocks in year three (Abdominal problems, Pulmonary and cardiac problems, Oncology, Nephrology and Endocrinology). The students' grades on these blocks were retrieved from the medical school data bank.

### Primary outcome

Students' grades on the end-of-block test served as the measure of the educational effectiveness of the formats. The test consisted of 10 multiple choice questions (maximum score 10 points), 20 extended matching questions (maximum score 40 points), and two open questions asking the students to use the standard analytical problem-solving method taught during the block to solve an orthopaedic problem (maximum score 23 points) and a rheumatologic problem (maximum score 23 points). All scores were summed (final score) and an overall grade was determined using the method of Cohen-Schotanus [24]. The sub-scores on the different types of questions as well as the overall grade were also included in the analysis.

### Secondary outcomes

#### Students' reasons for preferring one of the educational formats

Questionnaire A contained additional questions for the non-RCT participants, asking them to rate their agreement with statements concerning their preferences for aspects of education and studying on a 5-point Likert scale (strongly disagree, disagree, neutral, agree, strongly agree).

#### Satisfaction with the educational formats

Questionnaire B was emailed to all the study participants immediately after the end-of-block test, followed by one reminder to non-responders after two weeks. The students were asked to rate their agreement with statements concerning their satisfaction with the educational format and their opinion of the test on a 5-point Likert scale (strongly disagree, disagree, neutral, agree, strongly agree).

### Data analysis

The data of the non-RCT participants and the RCT participants were analyzed separately. We analyzed differences using student unpaired t test or chi-square test where appropriate. The analyses were based on intention-to-treat as initially assigned. Per protocol analysis was based on participation in at least two of the three sessions. The Statistical Package for the Social Sciences version 14.0 (SPSS, Chicago, IL) was used for all the analyses. P values of < 0.05 were considered statistically significant.

## Results

**Figure 1:**
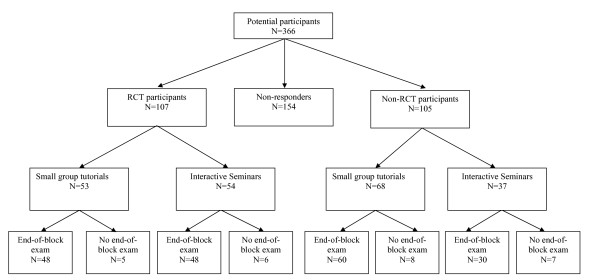
**Trial profile**. Legend figure 1. Potential participants = Students who were eligible to undertake the MusculoSkeletal Problems (MSP) block. RCT participants = Students who agreed to participate in the Randomized Controlled Trial. Non-RCT participants = Students who were free to choose between small group tutorials and interactive seminars and completed at least one of the questionnaires. Non-responders = Students who did not participate in the RCT and did not complete any of the questionnaires. End-of-block exam = End-of-block test on MusculoSkeletal Problems

Students were recruited during February 2007. A total of 366 potential participants (target population) in the MSP block were sent invitations to participate as well as questionnaires A and B (Figure 1). Of these students, 107 consented to participate in the RCT (RCT participants). They were randomly assigned to small group tutorials or interactive seminars, while 105 students did not participate in the RCT but provided data by completing at least one of the questionnaires (non-RCT participants).

### Baseline data

Questionnaire A was completed by 78 RCT participants (78/107; 73%) and 105 non-RCT participants (105/105; 100%) (Table 1). The majority of non-RCT participants who completed this questionnaire (68/105; 65%) chose to participate in small group tutorials. The RCT participants in the small group tutorials and the interactive seminars were similar with regard to gender, study hours, and grades on previous block tests. The non-RCT participants who attended the small group tutorials reported more study hours and had higher test grades compared with those preferring interactive seminars. These differences were not statistically significant, however. No differences were found between RCT and non-RCT participants in gender, study hours, and previous grades.

**Table 1:** Baseline characteristics of RCT and non-RCT participants by educational format.

	RCT		Non RCT		
	
	Small Group	Interactive Seminar	Small Group	Interactive Seminar	P value***
# students who completed questionnaire A	42	36	68	37	
		
Sex					
% Men	29	32	21	27	
					
Study hours*	%	%	%	%	
< 20hours	21.4	13.9	13.2	25.0	
21-30 hours	59.5	69.4	63.2	66.7	
> 30 hours	19.0	16.7	23.5	8.3	
					
Grades on block tests in Year 3**	Mean (SD)	Mean (SD)	Mean (SD)	Mean (SD)	
Abdominal problems	6.9 (0.9)	7.0 (1.0)	7.2 (1.0)	6.8 (1.2)	0.563
Pulmonary/cardiac problems	6.5 (1.2)	6.5 (1.3)	7.1 (1.5)	6.5 (1.3)	0.079
Oncology	6.4 (1.1)	6.4 (1.1)	7.1 (1.1)	6.7 (1.2)	0.228
Nephrology	6.2 (0.9)	6.2 (0.9)	6.9 (1.3)	6.7 (1.3)	0.492
Endocrinology	6.5 (1.1)	6.3 (0.9)	6.6 (1.3)	6.2 (1.4)	0.563

RCT = Students randomly assigned;

Non-RCT = Students who were free to choose between small group tutorials and interactive seminars;

Questionnaire A = Questionnaire sent to all students three weeks before the start of the block;

Study hours = hours/week spent on didactic lectures, small group tutorials and individual study;

Grades on a ten-point scale: 0 = lowest; 10 = highest;

* RCT: SG vs IS: P = 0.091; Non-RCT: SG vs IS: P = 0.086 both by chi-square test;

**all RCT vs all Non-RCT: P = 0.966 by chi-square test;

*** P value is a result of Student Unpaired t test and represents non-RCT participants: SG vs IS.

### Primary outcome

#### Educational effectiveness of the formats

Ninety-six (96/107; 90%) RCT participants and 90 (90/105; 86%) non-RCT participants sat the test. Their results are presented in Table 2. The RCT participants in the small groups and interactive seminars did not differ in overall test grades or sub-scores. In both groups, the mean overall grade was 6.6 (on a ten-point scale). Forty-one (41/48; 85%) of the participants in seminars and 42 (42/48; 87%) of the participants in small group tutorials passed the test, i.e. scored 6 or higher. The results of the per protocol analysis did not differ from the results of the intention-to-treat analysis (data not shown). The non-RCT participants who had chosen to attend small groups had significantly higher overall test grades and sub-scores compared to the non-RCT participants attending the interactive seminars.

**Table 2:** Effectiveness of small group tutorials and interactive seminars.

	RCT-participants				
	
	SG	IS	T-test		
		
Outcome	Mean (SD)	Mean (SD)	Mean Δ	95% CI	P-value
Test takers (N)	48	48			
End-of-block test grade (0-10; ≥6 = pass)	6.6 (1.1)	6.6 (1.0)	0.06	-0.36-0.48	0.768
Sub-scores (minimun vs maximum score):					
Multiple-choice questions (0-10)	6.3 (1.7)	6.2(1.4)	0.06	-0.58-0.70	0.846
Extended-matching questions (0-10)	25.7 (5.2)	25.8 (5.3)	-0.15	-2.28-1.97	0.887
Open questions orthopaedics (0-23)	15.0 (3.5)	14.1 (3.7)	0.92	-0.56-2.39	0.221
Open questions rheumatology (0-23)	15.9 (4.5)	15.5 (4.2)	0.41	-1.36-2.17	0.649
	**Non-RCT participants**				
	
	SG	IS	T-test		
	
	Mean (SD)	Mean (SD)	Mean Δ	95% CI	P-value
Test takers (N)	60	30			
End-of-block test grade (0-10; ≥6 = pass)	6.8 (1.2)	6.2 (1.3)	0.60	0.47-1.15	0.034
Sub-scores (minimun vsmaximum score):					
Multiple-choice questions (0-10)	6.3 (1.5)	5.7 (1.6)	0.52	0.15-1.18	0.126
Extended-matching questions (0-10)	27.1 (5.7)	25.2 (5.1)	1.91	0.54-4.36	0.125
Open questions orthopaedics (0-23)	15.3 (4.5)	13.0 (5.4)	2.32	0.18-4.46	0.034
Open questions rheumatology (0-23)	16.3 (4.5)	15.2 (4.8)	1.09	0.94-3.12	0.288

SG = Small group tutorials; IS = Interactive seminars.

End-of-block test grade = grade received at the final test.; sub-scores = number of points received for the different parts of the test.

Minimum vs maximum score: (min-max) For example: (0-10), minumum score = 0, maximum score = 10

### Secondary outcomes

#### Students' reasons for preferring one of the educational formats

Sixty-eight non-RCT participants (68/105; 65%) in small group tutorials and 37 non-RCT participants (36/105; 35%) in interactive seminars completed questionnaire A (Table 3). Compared with the participants in interactive seminars, significantly more participants in small groups stated a general preference for small group work (93% versus 64%; P = 0.001) and said they believed that participation in small group tutorials enhances retention of information (88% versus 44%; P = 0.000). The percentage of participants expressing a dislike of compulsory elements in teaching was higher among the participants in interactive seminars (56% versus 18%; P = 0.008).

**Table 3:** Non-RCT participants' reasons for preferring small groups or interactive seminars

	Non-RCT participants									
	
	SG					IS				
	
Number of students completing questionnaire A	N = 68					N = 36				
	
	strongly disagree	disagree	neutral	agree	strongly agree	strongly disagree	disagree	neutral	agree	strongly agree
	
	%					%				
In general, I prefer working in small groups.*	0	0	7.5	59.7	32.8	0	14.7	20.6	50.0	14.7
I retain information much better when I work in a small group.**	0	0	11.9	58.2	29.9	0	14.7	41.2	32.4	11.8
I want to participate actively during lectures and seminars.	0	9.0	20.9	47.8	22.4	0	17.6	26.5	41.2	14.7
I like to have the material demonstrated to me.	1.5	3.0	22.4	53.7	19.4	0	2.9	17.6	52.9	23.5
I appreciate direct feedback from a tutor/lecturer.	0	3.0	13.4	65.7	17.9	2.9	2.9	17.6	50.0	26.5
I dislike compulsory course components.***	1.5	37.3	43.3	9.0	9.0	8.8	8.8	26.5	41.2	14.7
I prefer to determine for myself when I study.	0	1.5	22.4	52.2	23.9	0	2.9	11.8	52.9	32.4
I prefer to work out the material for myself.	1.5	19.4	35.8	38.8	4.5	5.9	11.8	38.2	38.2	5.9
To me, small group teaching is an extra and not essential preparation for the exam.	16.4	55.2	4.5	19.4	4.5	11.8	41.2	20.6	26.5	0
I prefer to start studying just before the exam.	28.4	40.3	14.9	13.4	3.0	14.7	41.2	20.6	17.6	5.9
I enjoy preparing for small group tutorials.	3.0	13.4	26.9	50.7	6.0	5.9	14.7	29.4	38.2	11.8

SG = Small group tutorials. IS = Interactive seminars.

Questionnaire A = Questionnaire sent to all students three weeks before the start of the block.

* P = 0.001; ** P < 0.001 and *** P = 0.008 by student unpaired t test.

#### Satisfaction with the educational formats

Questionnaire B was completed by 83 RCT participants (83/107; 78%) and 95 non-RCT participants (95/105; 90%).

RCT-participants' opinions of the format they were assigned to are presented in Tables 4 and 5. Most of the students were satisfied with their tutors or lecturers (87% versus 86% in the small group tutorials and interactive seminars, respectively) and agreed that they had learned a great deal from the problems presented (87% and 84%, respectively). Forty-four percent of the participants in small group tutorials versus 47% of the participants in interactive seminars felt well prepared for the end-of-block test. Significantly more participants in small group tutorials than participants in interactive seminars expressed satisfaction with the educational format (86% versus 39%; chi-square; P < 0.001) and would again choose the format to which they had been randomized in this trial (56% versus 24%; chi square; P < 0.001). The responses of the non-RCT participants followed a similar pattern (data not shown). The percentage of non-RCT participants in interactive seminars who indicated they would choose the same format again was higher than that of the RCT participants in interactive seminars (44% versus 24%).

**Table 4:** Satisfaction with course format of RCT participants in small group tutorials

Number of students completing questionnaire B	45				

	
	**strongly** **disagree**	**disagree**	**neutral**	**agree**	**strongly** **agree**



**Questionnaire items**	%				

I was satisfied with the tutors.	2	4	7	71	16
I learned a lot from the problems presented in the course.	0	2	11	71	16
I felt well prepared for the exam.	36	0	20	40	4
I am satisfied with the course.	0	7	7	62	24
I would choose small group tutorials again next time.	2	20	22	25	31

Questionnaire B = Questionnaire sent to all students immediately after the end-of-block test.

**Table 5:** Satisfaction with course format of RCT participants in interactive seminars

Number of students completing questionnaire B*	38				
	

	**strongly** **disagree**	**disagree**	**neutral**	**agree**	**strongly** **agree**



**Questionnaire items**	%				

I was satisfied with the tutors.	0	3	11	68	18
I learned a lot from the problems presented in the course.	0	3	13	66	18
I felt well prepared for the exam.	3	32	18	42	5
I am satisfied with the course.	8	16	37	34	5
I would choose interactive seminars again next time.	18	50	8	21	3

Questionnaire B = Questionnaire sent to all students immediately after the end-of-block test

## Discussion

In order to shed light on the potential of interactive seminars as an alternative educational format to small group tutorials, we examined students' test results as a measure of the two formats' educational effectiveness, students' reasons for preferring small group tutorials or interactive seminars and students' satisfaction with the two formats. We found that small group tutorial leads to greater satisfaction but not to better learning results than interactive seminars.

Both formats proved to be equally effective as measured by the block test results. This applied to all test sub-scores including the two open questions which were specifically designed to assess students' ability to solve clinical problems. The only difference in test result was found among non-RCT participants: those who had opted for small group tutorial sessions outperformed those who had attended the interactive seminars. One possible explanation for this, partly based on the somewhat higher previous test grades of the students who chose to attend the small groups, may be that small group tutorial work is preferred by a select, highly motivated group of students, who are reluctant to allow their education to be determined by chance and who have a strong predilection for small group tutorials. The test results of the RCT participants, however, suggest that small group tutorials and interactive seminars presented by experienced teachers using audiovisuals are equally effective. Although the group sizes were quite large (48 each) it is possible that a type II error (there was a difference but the group size was too small to measure it) is present.

Given the choice, most students said they preferred small group tutorials, although there was a group of students for whom interactive seminars held a stronger appeal (37/105; 35%). We speculate that this choice may be based on a wish or need to use time economically. For participation in interactive seminars advance preparation is less mandatory.

The data provided by the students who decided freely (non-RCT) to participate in small group tutorials or interactive tutorials give us opportunity to speculate on the differences between the two groups of students. A preference for small group tutorials appears to be associated with a trend towards higher grades on previous tests, more time devoted to studying, a strong preference for small group work in general and higher expectations of its benefits, such as better retention of information, preparation for the exam, and easy access to direct feedback from an experienced teacher. The groups that opted for interactive seminars did somewhat less well on previous block tests, spent less time studying, showed less appreciation of small group work, and had a stronger aversion to obligatory study activities.

Although all respondents were satisfied with their teachers and the presentation of the problems, the students who took part in small group tutorials expressed greater satisfaction with the educational format and a stronger inclination to make the same choice again next time. The lower satisfaction with seminars than with small groups confirms findings from other studies that students and professionals generally prefer small group learning to learning in large groups [9,11,5].

The study design does not allow conclusions to be drawn with regard to the identification of students who are likely to benefit the most from either small group teaching or interactive seminars. The picture that emerges from the literature on the effectiveness of learning in small and large groups is not unequivocal. Our study is one of few studies that used the same measurement instrument to compare the educational effectiveness of different formats [9-11,25]. A study by Costa et al. [9] and by Doucet et al. [10] showed better knowledge retention after interactive teaching and better results from a PBL approach to continuing medical education, respectively. On the other hand, White et al. found no significant differences in knowledge uptake and retention when they compared a PBL and a didactic seminar format to disseminate guidelines among primary care physicians [11]. As a proxy for the comparison between teaching in small and in large groups, we looked at studies comparing problem-based medical curricula and traditional lecture-based curricula. Conventional measurements of knowledge showed no large effects in favour of problem-based learning [19,26] but they did suggest a positive effect of problem-based learning on students' satisfaction and ability to solve clinical problems [27]. This seems to be consistent with the results of our study.

Our study has several limitations that affect the generalizability of the findings.

To start with, due to ethical reasons, we have no information on the non-responders (154/366; 42%). Also, the non-RCT participants' response to the questionnaires was lower than that of the RCT participants, thus limiting the amount of information about the non-randomized group. Secondly, participants' exposure to the two educational formats was limited to three two-hour sessions. In addition, to provide data, students were allowed to miss one of the three sessions thus further limiting the amount of exposure to the educational format. It should also be noted that participation in small group tutorials may affect other competencies besides cognition, which was predominantly measured by the block test. This suggests that the educational impact of the two formats may differ when other competencies are taken into account as well. Moreover, it must be stressed that the small group tutorials in our study differed from 'traditional' PBL tutorials. The deviations from the original design such as much larger group of participants and different built-up of the problem-solving might explain why we were unable to demonstrate the impact described with the 'traditional' PBL tutorials. Finally, the success of the two formats depends largely on teachers' experience in interactive teaching and therefore cannot be transferred to other settings. This implies that it is important to study the implementation of interactive seminars as an alternative to small group tutorials in different settings.

## Conclusion

Although the students were more satisfied with the small group tutorials, about one-third preferred interactive seminars, which accommodate larger groups, when given a choice. Since the results showed no negative effects of this choice on test results, it might be worthwhile to conduct further studies to investigate whether and how interactive seminars can offer an acceptable, more cost effective alternative to small group tutorials.

## Competing interests

The authors declare that they have no competing interests. The authors alone are responsible for the content and writing of this article.

## Authors' contributions

ZdJ has made a substantial contribution to the conception and design, analysis and interpretation of data, and has been involved in drafting the manuscript. JvN has made a substantial contribution to the acquisition, analysis and interpretation of data and has been involved in drafting of the manuscript. SP has made a substantial contribution to the acquisition, analysis and interpretation of data and has been involved in drafting of the manuscript. SV has made a substantial contribution to the conception and design, and has been involved in revising the manuscript for important intellectual content. FD has made a substantial contribution to the analysis and interpretation of the data and has been involved in revising the manuscript for important intellectual content. AS has made a substantial contribution to the conception and design and has been involved in drafting the manuscript.All authors read and approved the final manuscript.

## Authors'information

Zuzana de Jong, MD, PhD, is a rheumatologist and a coordinator of the block MusculoSkeletal Problems, Faculty of Medicine, Leiden University Medical Center (LUMC), Leiden, the Netherlands.

Jessica van Nies is a sixth-year medical student at LUMC.

Sonja Peters is a sixth-year medical student at LUMC.

Sylvia Vink, MA is an educational advisor and a teacher trainer at Leiden University, Leiden.

Friedo Dekker, PhD, is a clinical epidemiologist and head of the Center for Educational Expertise in Medicine at LUMC.

Albert Scherpbier, MD, PhD, is professor of Quality Assurance in Medical Education and scientific director of the Institute for Medical Education, Faculty of Health, Medicine and Life Sciences, Maastricht University, Maastricht, the Netherlands.

## Pre-publication history

The pre-publication history for this paper can be accessed here:

http://www.biomedcentral.com/1472-6920/10/79/prepub
